# Complete mitochondrial genome of *Pseudoglomeris magnifica* (Shelford, 1907) (Insecta: Dictyoptera: Blaberidae)

**DOI:** 10.1080/23802359.2022.2119823

**Published:** 2022-09-15

**Authors:** Yu Bai, Kang Yang, Lin Ye, Xuyuan Gao

**Affiliations:** a College of Mathematics & Information Science, Guiyang University, Guiyang, China; b College of Biology and Environmental Engineering, Guiyang University, Guiyang City, China; c Institute of Plant Protection, Guangxi Academy of Agricultural Sciences, Guangxi Key Laboratory of Biology for Crop Diseases and Insect Pests, Nanning, China; d Ministry of Agriculture and Rural Affairs, Institute of Plant Protection, Guangxi Academy of Agricultural Sciences, Key Laboratory of Green Prevention and Control on Fruits and Vegetables in South China, Nanning, China

**Keywords:** *Pseudoglomeris magnifica*, Dictyoptera, *Corydidarum*, mitochondrial genome, mitogenome

## Abstract

*Pseudoglomeris magnifica* (Shelford, 1907) is bred as a pet because of its beautiful appearance. Its complete mitochondrial genome (GenBank accession number MW630139), obtained from the Manwan Town population, was characterized as the first complete mitogenome of the genus *Pseudoglomeris*. The mitogenome consists of a circular DNA molecule of 16,627 bp with 76.23% AT content. It comprises 13 protein-coding genes (PCGs), 22 tRNA genes, two rRNA genes, and one control region. The PCGs have the traditional ATN (Met) start codon, except *cox1* and *nad1* (which have TTG (Met) as the start codon), and are terminated by the traditional TAN stop codon.

The genus *Pseudoglomeris* belongs to the order Blattodea, superfamily Blaberoidea, family Blaberidae, and subfamily Perisphaerinae. This genus has five new junior synonyms: *Corydidarum*, *Trichoblatta*, *Kurokia*, *Glomerexis*, and *Glomeriblatta* (Li et al. 2018). *Pseudoglomeris magnifica* (Shelford, 1907) (Shelford 1907) is bred as a pet because of its beautiful appearance and its complete mitochondrial genome obtained from the Manwan Town population was characterized in this study as the first complete mitogenome of the genus *Pseudoglomeris* (Figure 1).

**Figure 1.**
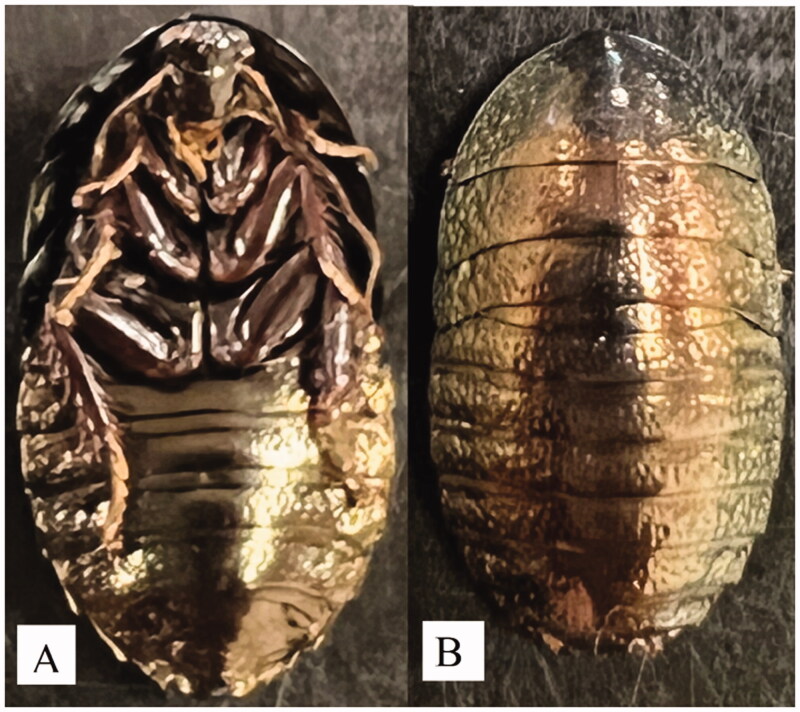
Species reference image of *Pseudoglomeris magnifica*. (A) The ventral view of *P*. *magnifica*; (B) the dorsal view of *P*. *magnifica.*

Adult *P. magnifica* specimens were collected from Shuibatou Village (100.361795 N, 24.703026 E), Manwan Town, Yun County, Lincang City, Yunnan Province, China on 13 September 2020 and deposited in the Insect Collection of the Institute of Plant Protection, Guangxi Academy of Agricultural Sciences (http://www.gxaas.net/s.php/zwbhyjs/, Xuyuan Gao, gxy@gxaas.net) under the voucher number GIPP-20200913-001. Genomic DNA was isolated and subjected to paired-end sequencing (2 × 150 bp) of 350 bp inserts using the Illumina NovaSeq 6000 platform (Illumina, San Diego, CA) according to the manufacturer’s instructions. We obtained approximately 10.0036 Gbp of raw data (66,690,850 reads), of which 9.8994 Gbp (98.96%, 66,398,126 reads) was high-quality, and cleaned the data using cutadapt v1.9.1. The mitogenome was assembled *de novo* using Velvet 1.2.10 (Zerbino and Birney 2008), and gaps were filled with SSPACE 3.0 (Boetzer et al. 2011) and GapFiller 1.1 (Boetzer and Pirovano 2012). The 158,836 mitochondrial reads (0.24% clean reads) defined mitogenome of *P. magnifica* (GenBank accession number: MW630139) with 100% mitogenome coverage at high average reads depth (823.91 times).

The mitogenome of *P. magnifica* consisted of a circular DNA molecule of 16,627 bp (40.78% A, 35.45% T, 14.80% C, and 8.97% G; 76.23% AT content). Using Perna and Kocher’s formula (Perna and Kocher 1995), the AT and GC skews of the major strand of the mitogenome were estimated to be 0.0698 and −0.2449, respectively.

The mitogenome of *P. magnifica* contains 13 protein-coding genes (PCGs) annotated using MITOS 2.0.6 (Bernt et al. 2013), 22 tRNA genes detected using tRNAscan-SE (Chan and Lowe 2019), and two rRNA genes identified using RNAmmer (Lagesen et al. 2007). The order and orientation of the genes were determined and drawn (Figure 2) using the OGDRAW web server (https://chlorobox.mpimp-golm.mpg.de/OGDraw.html) (Greiner et al. 2019). The control region (CR, also an AT-rich region) in the mitogenome is 1338 bp in length, with 80.04% AT content, and is located between the genes encoding srRNA and tRNA-Ile. Eleven PCGs (*nad3*, *atp8*, *cox2*, *nad6*, *cob*, *nad4l*, *nad4*, *cox3*, *atp6*, *nad5*, and *nad2*) had a traditional ATN (Met) start codon, whereas *cox1* and *nad1* had a TTG start codon. One gene (*atp8*) started with an ATA start codon, seven genes (*nad2*, *cox2*, *atp6*, *cox3*, *nad4*, *nad4l*, and *cob*) started with an ATG start codon, and three genes (*nad3*, *nad5*, and *nad6*) started with an ATT start codon. All 13 PCGs had traditional TAN stop codons. Eleven genes (*cob*, *nad4l*, *nad5*, *cox3*, *atp8*, *nad4*, *cox2*, *nad3*, *atp6*, *nad2*, and *nad6*) ended with a TAA stop codon, one gene (*nad1*) ended with a TAG stop codon, and one gene (*cox1*) had an incomplete stop codon consisting of a T, which is known to be completed by the addition of A nucleotides to the 3′ end of the encoded mRNA. The 22 tRNAs were interspersed throughout the PCGs, with lengths ranging from 61 bp (tRNA-Cys) to 73 bp (tRNA-Tyr). The 16S and 12S rRNA were 1293- and 782-bp long, respectively. The maximum overlap, with a length of 15 bp, was observed between tRNA-Trp and tRNA-Cys.

**Figure 2.**
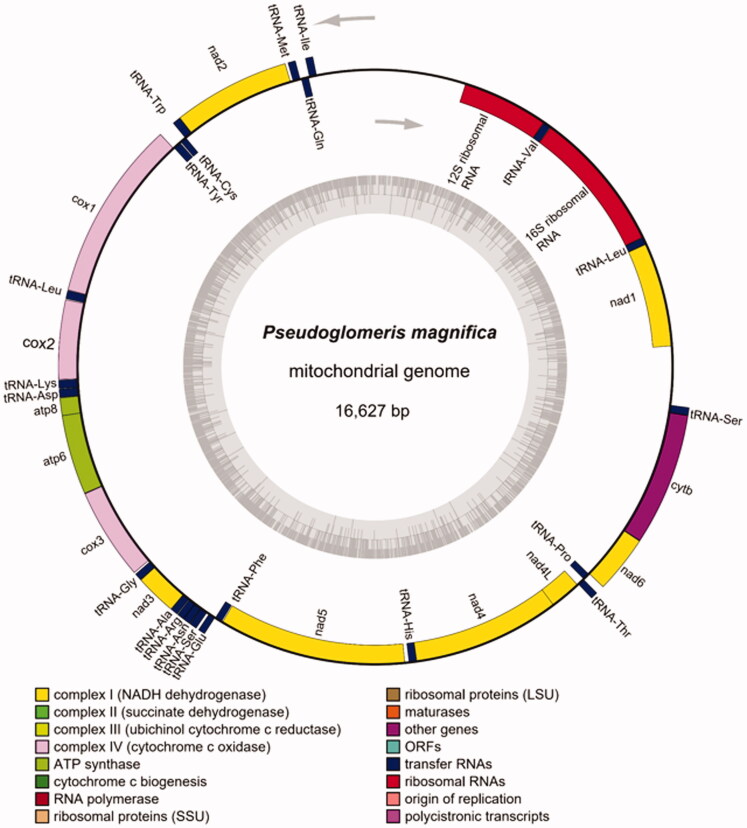
Mitogenome pattern map of *Pseudoglomeris magnifica.*

For phylogenetic analyses of *P. magnifica*, their 13 mitochondrial PCGs and those of nine other species of Insecta were used to construct a phylogenetic tree using MEGA X software (Kumar et al. 2018) via the maximum-likelihood method (Figure 3). Seven mitogenomes were obtained from the superfamily Blaberoidea. The *Lepisma saccharina* (Bai et al. 2020), *Tenebrio obscurus* (Bai et al. 2018), and *Promethis valgipes valgipes* (Bai et al. 2021) mitogenomes were selected as the outgroup. The amino acid sequences of 13 PCGs of each mitogenome were connected end to end in the order of *nad2*-*cox1*-*cox2*-*atp8*-*atp6*-*cox3*-*nad3*-*nad5*-*nad4*-*nad4l*-*nad6*-*cob*-*nad1* to form a sequence. The protein sequences of the PCGs in their mitogenomes were aligned using MEGA X software (Kumar et al. 2018) with the MUSCLE program (Edgar 2004) using default specifications. The ML model with the lowest Bayesian information criterion (BIC) scores was considered to be the best. Based on BIC (=86750.255), general reversible mitochondrial model (mtREV24) with amino acid frequencies (+F)+gamma distribution (G) (parameter = 0.5107) with five rate categories was chosen as the optimal evolutionary model with 1000 bootstrap replications for phylogenetic analysis. The phylogenetic position of *P. magnifica* within the superfamily Blaberoidea was first evaluated using complete mitogenomes, and the structure of the phylogenetic tree is similar to reported in previous studies (Roth 2003). The accurate phylogeny within the superfamily Blaberoidea will require more mitogenomes, because of the limitations of the current mitogenomes. The newly sequenced complete mitogenome of *P. magnifica* will contribute to the preservation of its natural resources and the phylogenetic analysis of this species.

**Figure 3.**
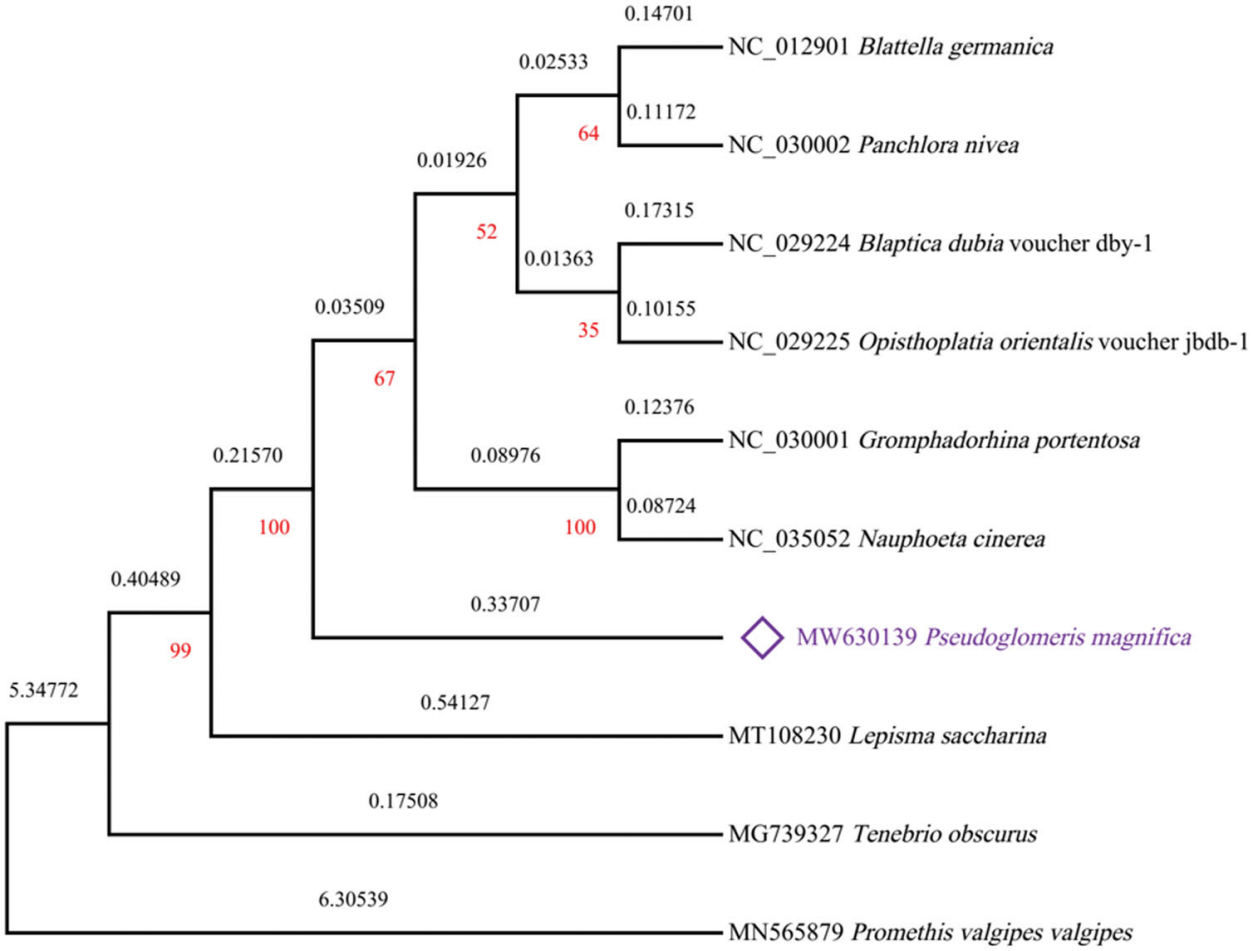
Maximum-likelihood phylogenetic tree of *Pseudoglomeris magnifica* and nine other Insecta based on protein sequences on 13 PCGs of their mitogenomes.

Phylogenetic relationships were inferred using maximum-likelihood method and General Reversible Mitochondrial + Freq. model + A discrete Gamma distribution (+G, parameter = 0.5107). The tree with the highest log likelihood is –43176.91. The percentage of trees in which the associated taxa clustered together is shown in red numbers below the branches. The black numbers above the branches indicated branch lengths which measured the number of substitutions per site. The complete mitogenome of *P. magnifica* from this study was indicated in purple color.

## Author contributions

Yu Bai analyzed the data, uploaded the analysis data, involved in certain tools for analysis, drafted of the paper, and approved the final draft. Kang Yang performed the experiments. Lin Ye collected and analyzed data. Xuyuan Gao identified insects, contributed reagents/materials, involved in conception and design of the work, performed the experiments, prepared figure, and approved and published the final draft. All authors agree to be accountable for all aspects of the work.

## Ethics statement

This research does not involve ethical research. Insects are invertebrates, and there are no ethics involved in using them in experiments.

## Data Availability

The genome sequence data that support the findings of this study are openly available in GenBank of NCBI at https://www.ncbi.nlm.nih.gov under the accession no. MW630139. The associated BioProject, SRA, and Bio-Sample numbers are PRJNA702610, SRR13743950, and SAMN17976222, respectively.
